# Epigenetic mechanisms in sex determination and in the evolutionary transitions between sexual systems

**DOI:** 10.1098/rstb.2020.0110

**Published:** 2021-08-30

**Authors:** Francesc Piferrer

**Affiliations:** Institut de Ciències del Mar (ICM), Spanish National Research Council (CSIC), Passeig Marítim, 37-49, 08003 Barcelona, Spain

**Keywords:** DNA methylation, genetic assimilation, sexual system, sex-determining mechanisms, sex chromosomes, Williams' paradox

## Abstract

The hypothesis that epigenetic mechanisms of gene expression regulation have two main roles in vertebrate sex is presented. First, and within a given generation, by contributing to the acquisition and maintenance of (i) the male *or* female function once during the lifetime in individuals of gonochoristic species; and (ii) the male *and* female function in the same individual, either at the same time in simultaneous hermaphrodites, or first as one sex and then as the other in sequential hermaphrodites. Second, if environmental conditions change, epigenetic mechanisms may have also a role across generations, by providing the necessary phenotypic plasticity to facilitate the transition: (i) from one sexual system to another, or (ii) from one sex-determining mechanism to another. Furthermore, if the environmental change lasts enough time, epimutations could facilitate assimilation into genetic changes that stabilize the new sexual system or sex-determining mechanism. Examples supporting these assertions are presented, caveats or difficulties and knowledge gaps identified, and possible ways to test this hypothesis suggested.

This article is part of the theme issue ‘Challenging the paradigm in sex chromosome evolution: empirical and theoretical insights with a focus on vertebrates (Part I)’.

## 1. Introduction

To explain how epigenetics can contribute to sex determination and to the evolutionary transitions between different sexual systems, first it is necessary to briefly define some terms used throughout the text, as hermaphroditism, one of several types of sexual systems, has been regarded as epigenetic sex determination (e.g. [1]). Next, examples concerning the increasing evidence for a role of epigenetics in sex determination, maintenance and plasticity in different taxa will be presented. Finally, the idea that epigenetics participates in the evolutionary transitions between different sexual systems and between different sex-determining mechanisms will be discussed.

### (a) Sexual systems

To describe sex, two scales of classification have traditionally been used: (i) the sexual system, and (ii) the sex-determining mechanism (see §1b below). A sexual system, according to Leonard [2], is defined as the pattern of sex allocation that exists among individuals of a given species. In general, the two most stable and abundant sexual systems, both in plants and animals, are dioecy (gonochorism) and hermaphroditism. In the former, male (sperm production) and female (egg production) functions are separated in different individuals, while in the latter male and female functions take place in the same individual, either simultaneously or sequentially. Sequential hermaphrodites reach adulthood and sexually mature first as one sex. Then, the gonads experience extensive tissue reorganization and the animal stops producing gametes of the first sex and starts producing gametes of the opposite sex. This process is termed sex change. However, there are mixed systems such as androdioecy (species consisting of males and hermaphrodites), that are less stable and thus less widely distributed [2,3].

Sexual systems in organisms can be viewed as exhibiting different degrees of phenotypic plasticity, with gonochorism and genetic sex determination at one end (e.g. mammals, birds and many insects), having a minimum of sexual plasticity; and, at the opposite end, simultaneous hermaphroditism (e.g. some fishes, many different types of invertebrates), with a maximum of sexual plasticity [2,4]. Transitions between different sexual systems are common and have been documented in different groups of vertebrates [2,5]. Leonard [2] argued that evolutionary transitions between different sexual systems, specifically between gonochorists and simultaneous hermaphrodites, would occur through intermediate stages consisting of species with environmental sex determination (ESD) or sequential hermaphroditism.

Based on what has been discussed so far, it seems clear that phenotypic plasticity is an inherent property in the diversity of sexual systems and is necessary for the evolutionary transitions among them. As we will see below, epigenetics underlies phenotypic plasticity.

### (b) Sex-determining mechanisms

Sex-determining mechanisms can be classified into two major types according to the nature of the main sex-determining factor: (i) genotypic sex determination (GSD), where the factor is genetic, with different mechanisms (chromosomal, polygenic); and (ii) ESD, where the main factor is an environmental cue. In ESD, at least in fishes [6] and reptiles [7], the temperature is the most common environmental factor (temperature-dependent sex determination, TSD). However in many species, sex determination actually depends on the contribution of both genetic and environmental factors [8–11]. Nowadays, the sex-determining mechanism is viewed across a gradient of possibilities in which ‘pure’ GSD and ESD species just represent opposite ends of this continuous gradient, with many possible intermediate combinations [12].

**Figure 1.**
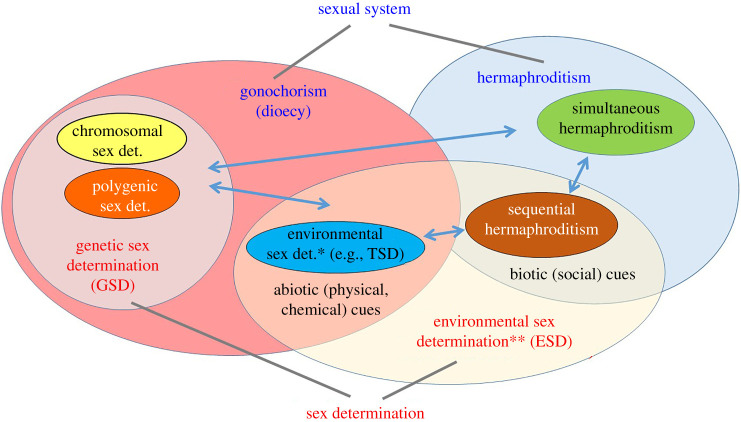
Diagram representing the integration of sexual systems and mechanisms of sex determination in a common framework. The different states are represented as discrete although in fact there is a continuum gradient of possible, intermediate, states. The idea of the evolutionary transitions along a gradient of phenotypic plasticity was postulated by [2,4]. Epigenetic mechanisms underlie and made possible this phenotypic plasticity. Notice that environmental sex determination is mentioned both *sensu stricto* (*) and *sensu lato* (**). See §1b. Figure is redrawn and updated from Piferrer [15].

It may be argued that hermaphrodites can be regarded as a form of ESD. However, simultaneous hermaphrodites develop gonads with male and female function taking place at the same time or within a short period of time, so ESD controlling primary sex determination here is problematic, or at least should not be considered as done for gonochoristic species because all individuals develop gonads having both male and female tissues. It may be argued that in the same way that sex determination in a GSD gonochoristic species takes place in the gonads of individuals of a population, in simultaneous hermaphrodites sex determination takes place at the gonadal level between the male and female parts of the gonads of an individual. On the other hand, in sequential hermaphrodites, individuals of a given species typically first reproduce as females (protogynous species) or as males (protandrous species) and hence the first sex seems also genetically established. However, the genetic factors responsible for primary sexual development and the underlying molecular mechanisms are poorly understood, although based on current evidence it is safe to assume that they use the same toolkit and gene networks involved in sexual development in gonochoristic species [13]. The influence of the environment in primary sexual development in hermaphrodites is, in contrast, plausible in protogynous diandric (two types of males: males can differentiate either directly or from females through sex change) and protandrous digynic (two types of females: females differentiate either directly or from males through sex change) species [2]. For example, in the diandric wrasse, *Halichoeres poecilopterus*, terminal phase (TP) males are large territorial males with bright body coloration and are derived either from initial phase (IP) females that change sex to male or from IP primary males that change colour and behaviour, but do not change sex. By performing cohabitation experiments involving different types of fish, it was found that TP transition in primary males was related to a dominance relationship (or size order) within social groups [14]. Aside from these diandric or digynic species, in sequential hermaphrodites, then, what is usually environmentally controlled is not the first sex that differentiates but, rather, the process of sex change, which can be regarded as a trans-differentiation of the adult gonad from the production of one type of gametes to the opposite type. Sex change in sequential hermaphrodites can then be environmentally controlled, but the environmental factor is usually biotic or social (presence of dominant conspecifics, population density, population sex ratio) [13], and thus different from the abiotic factors such as temperature controlling sex determination in gonochoristic species with ESD. In sequential hermaphrodites, be it either protogyny (female-to-male sex change) of protandry (male-to-female sex change), there is a complete reorganization of the gonadal tissues [13]. With these considerations made, figure 1 provides a picture that combines sexual systems and sex-determining mechanisms under the same framework.

There is abundant literature on the evolution of sex chromosomes and new sex-determining mechanisms. Models include genetic drift, pleiotropic selection of sex-determining genes, sex ratio selection and sexually antagonistic selection [5,16–20] (see also the paper by Perrin in this volume [21]). Thus, transitions between different mechanisms of sex determination are not only common but, importantly, can take place in a relatively short time. For example, turnover in the sex chromosomes and the sex-determining gene in medaka, *Oryzias latipes*, and its closely related species can occur in short evolutionary times [22]. The divergence of *Oryzias curvinotus*, which has *dmY* as its sex-determining gene, from *Oryzias luzonenzis*, which has *gsdf*, was calculated to occur approximately 10 million years ago [23]. If sex reversal (i.e. animals with a sexual phenotype opposite to their sex chromosome constitution) is brought in, then changes from GSD to TSD is possible in a single generation, as shown in the central bearded dragon, *Pogona vitticeps*, in which elevated temperatures sex reverse ZZ males into functional females eliminating the W chromosome and becoming TSD [11].

It has been argued that current models do not take into account the underlying developmental mechanisms, and that the increasing availability of molecular data will help to clarify how selection and developmental architecture interact to direct the evolution of sex-determination genes [19]. Epigenetics, in addition to phenotypic plasticity, also underlies cell fate commitment (as in sex determination and differentiation) and tissue reorganization (as in sex change, and possibly also in sex reversal [24]). Thus, the following section presents examples of the accumulating evidence for the role of epigenetic regulatory mechanisms in sex acquisition, maintenance and plasticity.

## 2. Epigenetics and sex determination and differentiation

### (a) Brief introduction to epigenetics

Epigenetics involves a set of chemical modifications either directly to the cytosine bases of DNA or to the histone proteins that constitute the chromatin and package the genome. The main epigenetic mechanisms are DNA methylation and histone modifications (methylation, acetylation, etc.) and variants. These modifications influence how genes are expressed by regulating chromatin structure and DNA accessibility [25]. Epigenetic regulatory mechanisms have a key role in controlling gene expression across a diverse array of developmental stages, tissue types, physiological states and environmental signals [26]. Epigenetic changes can be inherited not only during mitosis from mother to daughter cells but also through meiosis from parents to offspring, thus contributing to the transmission of acquired states of gene expression during cell and tissue differentiation within and across generations [27]. Furthermore, epigenetic mechanisms are susceptible to environmental change and thus have a major role in integrating genomic and environmental information to bring about the phenotype [28,29]. Species with sexual lability, and hermaphrodites in particular, constitute clear examples of phenotypic plasticity as both sexes can be produced from the same genotype. Thus, hermaphroditism has been considered as the product of an epigenetic sexual determination system [1].

### (b) Epigenetics and sex chromosome evolution

The implication of epigenetics on sex chromosome evolution was first proposed by Gorelick [30] who argued that initial differences between sexes (without focusing on any particular taxa) are determined by differential methylation in nuclear DNA between females and males, driving Muller's ratchet. The same principle has also been proposed for the situation in mammals, with male heterogamety [31], and in birds, with female heterogamety [32]. According to this view, differences in methylation of sex chromosomes lead to recombination suppression, hence increasing mutation rate and further accelerating the speed of Muller's ratchet [30]. The evolution of sex chromosomes via methylation is challenging to test, as recently discussed by Furman *et al*. [33], for three main reasons: (i) because the relationship between DNA methylation and gene expression can be dependent on the genomic region being considered (e.g. [34,35]); (ii) the fact that methylation regulation can occur in *trans*, i.e. it may affect distant loci with enhancer or repressor activity; and (iii) that methylation of specific loci may be erased during gamete formation [33]. Nevertheless, using whole genomic bisulfite sequencing (WGBS), different DNA methylation profiles between the sex chromosomes have been found, for example, in the half-smooth tongue sole, *Cynoglossus semilaevis* (ZW/ZZ system) [36,37], and in the three-spine stickleback, *Gasterosteus aculeatus* (XX/XY system) [38]. Taken together, these observations clearly indicate sex-related differences in DNA methylation of sex chromosomes but whether these differences are a cause or a consequence of sex chromosome differentiation still remains to be elucidated.

### (c) Epigenetics and sex fate commitment

In the last years, evidence has been accumulating on the implication of epigenetics in regulating the expression of key genes involved in sexual development, from plants to animals [39]. The rest of this section will deal with examples of the epigenetic differences between some of those genes, focusing on vertebrates.

The involvement of epigenetic regulatory mechanisms in sexual development is now supported by studies conducted in both GSD and ESD species in different taxa. The first evidence linking temperature to gene expression via epigenetics in vertebrates was obtained in the European sea bass, *Dicentrarchus labrax* (polygenic sex determination, PSD), where elevated temperature results in an increase in the number of males. This was related to hypermethylation of the promoter of gonadal aromatase (*cyp19a1a*), the enzyme responsible for oestrogen synthesis, and concomitant transcriptional downregulation of *cyp19a1a* [40]. Subsequent work on the same species showed that lasting effects of elevated temperature involved alterations in the expression of genes involved in a different type of epigenetic regulation including euchromatic histone-lysine N-methyltransferase 2 (*ehmt2*), the histone demethylase Jumonji (*jarid2a*) and polycomb group ring finger 2 (*pcgf2*) [41]. In the half-smooth tongue sole (ZZ/ZW), elevated temperature resulted in hypomethylation of the *dmrt1* promoter, leading to masculinization of ZW females, a change that was inherited in the unexposed ZW offspring, i.e. spontaneous sex reversal without environmental stimuli [37]. In this species, *dmrt1* is involved in sex determination [36]. These results provide evidence of a common mechanism regulating GSD and ESD and their coexistence in the same species.

In the red-eared slider turtle, *Trachemys scripta*, similar findings were made regarding the *cyp19a1* promoter, which was also hypomethylated in female-promoting temperature (FPT) [42]. Interestingly, FPT in turtles are elevated when compared to male-producing temperatures (MPTs), whereas the situation is reversed in fishes [6]. Nevertheless, in both fishes and reptiles, MPTs consistently involve hypomethylation of *cyp19a1a*. This suggests that signal transduction of the initial cue (elevated temperature) can have different outcomes between turtles and fishes. Subsequent work in *T. scripta* confirmed previous findings and showed an association of promoter region hypomethylation with canonical transcriptional activation markers, H3K4me3 and RNA polymerase II [43], indicating multi-layer epigenetic modifications in the regulation of sexual development. It was also demonstrated that *dmrt1* has a temperature-dependent, sexually dimorphic expression pattern, that is both necessary and sufficient to initiate male development in *T. scripta*, and that DNA methylation dynamics of its promoter were also correlated with temperature, suggesting that *dmrt1* is a candidate master male sex-determining gene in this TSD species [44]. Furthermore, the temperature is able to increase the transcription of lysine-specific demethylase 6B (*kdm6b*)*,* a chromatin modifier gene that eliminates the trimethylation of H3K27 in the promoter of *dmrt1*, leading to the upregulation of its expression and male development [45,46] (see also the paper by Weber & Capel [47] in this volume). In mice, *Jmjd1a*, a H3K9 demethylase, controls the expression of the mammalian Y chromosome sex-determining gene *Sry* by regulating H3K9me2 marks [48]. Sex reversal is common in some species of rodents. In the Akodon grass mice, *Akodon azare*, some XY males with an intact Y chromosome with a non-mutated *Sry* gene develop as fertile females and the underlying cause is the result of epigenetic modifications in sex chromosomes [49]. Thus, epigenetic regulatory mechanisms are also involved in mammalian sex determination [50].

### (d) Epigenetics, sex and mechanistic models

Based on the current knowledge on the role of epigenetics in sexual development, some mechanistic models have recently been proposed. Analysing data gathered in more than a dozen different species of fishes, including both gonochoristic and hermaphroditic species, Piferrer [51] and Piferrer *et al*. [52] proposed the model of the conserved epigenetic regulation of sex (CERS). This model, based on the regulation of gene expression by DNA methylation [34], contemplates sex-specific differences in DNA methylation and expression levels in genes involved in sexual development and, importantly, that these sex-specific differences are conserved regardless of taxa or the sexual system, i.e. whether one considers gonochoristic or hermaphrodite species. Thus, patterns of DNA methylation and gene expression are similar for primary male sex differentiation in gonochoristic species and for protogynous (female-to-male) sex change in hermaphrodite species because in both cases the end result is the same: a testis. Likewise, patterns are similar for female sex differentiation and protandrous sex change (male-to-female) with an ovary as the end result. Of note, the pattern is particularly consistent for key genes in the sexual development network such as *dmrt1* and *cyp19a1a*, and thus CERS probably can also be applied to reptiles based on the findings discussed earlier, while for other genes such as *amh*, *foxl2*, *sox9* and *gsdf*, data in additional species is needed [52].

As stated above, CERS is based on data collected mostly from gonochoristic fish species but also from a few hermaphrodites and is also compatible with findings so far in reptiles. Recently, further evidence for distinct epigenetic reprogramming and the involvement of the stress axis has been gathered concerning environmentally induced sex plasticity in vertebrates in general [53] and sex change in fishes in particular [54,55]. The emerging picture is that epigenetic modifications constitute a critical link between environmental stimuli, the onset of sex change in hermaphrodites, and subsequent maintenance of the new sexual phenotype [56].

Regarding the proximal sensor of environmental stimuli, it has been proposed that environmental cues are sensed through conserved elements of calcium and redox status that are transduced to cellular signal pathways, and/or influence epigenetic processes, to ultimately drive the differential expression of sex genes (the CaRe model) [57]. If proved correct, this would provide information on how cues such as temperature are transduced. However, it could also be tested in other circumstances, e.g. cues such as population density or population sex ratio driving sex change in sequential hermaphrodites. In any case, the CERS and CaRe models are compatible and complementary because in fact they concern different parts and aspects of the pathway, from the initial environmental cue to the ultimate sexual fate. Histone modifications may be induced before methylation changes that then serve as more stable epigenetic marks [58]. The proposed mechanistic models linking environmental signals and sex-determination pathways can be useful to direct further research.

Most of the examples above concern DNA methylation and histone modifications but there is also evidence on long-term changes in miRNA expression—also considered a type of epigenetic regulation of gene expression—in response to environmental cues [59]. Taken together, the findings discussed above provide mounting evidence for the involvement of epigenetic regulatory mechanisms in sex chromosome evolution and in sex determination and differentiation across different taxa. Next, the implication of epigenetics for evolutionary transitions will be discussed.

## 3. Epigenetics as a hub for evolutionary transitions

### (a) Epigenetics and phenotypic responses to environmental variation

**Figure 2.**
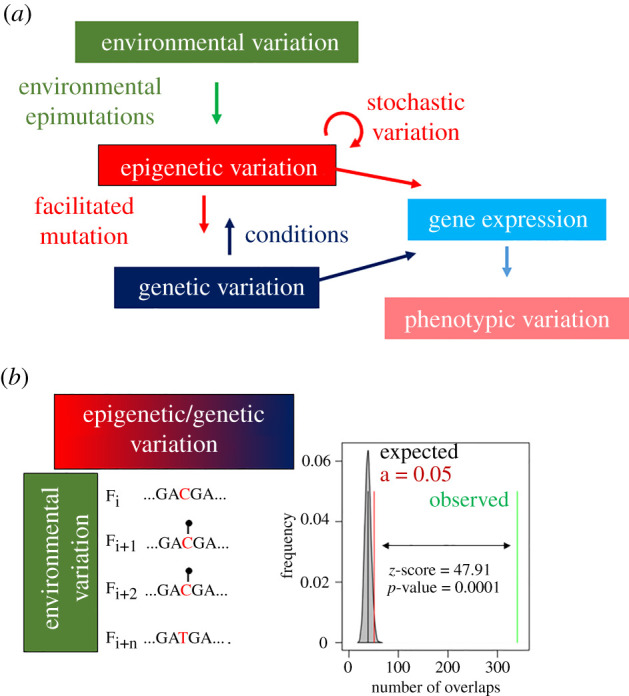
Epigenetic variation as a link between genetic variation and environmental variation that ultimately influence phenotypic variation. (*a*) General model. Environmental variation induces epimutations contributing, in addition of stochastic epimutations, to epigenetic variation. If the direction of environmental change persists in a sufficient number of generations, epimutations may end up assimilated as genetic variants. Both genetic and environmental variation (through epigenetics) influence gene expression and, in turn, phenotypic variation. (*b*) Mechanistic model by which persisting environmental variation across generations (*i*, …, *i* + *n*) can alter methylation (lollipop) of CpG loci (left) and actual association of environmentally induced epimutations, differentially methylated cytosines (DMCs) in this case, with on-the-spot single nucleotide polymorphisms (SNPs) in two sea bass populations (right). The number of overlaps of the two genomic sites is shown. The shaded grey area shows the number of overlaps of randomized regions with the mean represented by the black bar. The green line represents the actual number of overlaps of SNPs with DMCs and the double arrow its distance from the significance limit in red. The significance of the association is indicated by the *z*-score and the *p*-value (modified from [63]).

Epigenetic mechanisms can respond to environmental variation and facilitate phenotypic plasticity [28,29]. Novel phenotypic variants generated by epigenetic modifications in response to environmental change increase the evolutionary potential of a population because they promote genetic adaptation by different means [60]. In addition, epigenetic modifications allow for more rapid phenotypic responses to novel environments than are possible via the accumulation of genetic variation [61]. Thus, it is well established that epigenetic variation is one of the most important contributors to phenotypic variation in a population [62]. The causal relationships between genetic, environmental, epigenetic and phenotypic variation are shown in figure 2*a*.

### (b) Epigenetics and evolution

The possibility that an epigenetic modification might give rise to a localized change in DNA sequence, thereby converting an epigenetic into a genetic change, has been contemplated for some time. Further, it has been argued that this chain of events constitutes a possible route through which the environment might directly influence evolution, provided the induced genetic change has phenotypic effects on which selection can act [28,58]. Empirical evidence supporting this possibility is being gathered in recent years.

Danchin *et al*. [64] reviewed the current knowledge on the role of epigenetics in favouring evolutionary change and proposed that epigenetics is a sort of hub for evolutionary transitions. The main idea is that when environmental changes remain stable for a sufficient number of generations, information inheritance systems gradually move from ones that are relatively labile to more faithful and persistent ones that, of note, can be set as genetic variants [64]. This idea mainly concerns DNA methylation and is based, among other aspects, on the well-known hypermutability of methylated cytosine residues, susceptible to deamination and with a higher rate of change to thymine (epimutations) than non-methylated bases [64]. Because of their nature, these ideas are difficult to test and thus alternatives must be explored.

Domestication can be regarded as just one particular type of driving forces of evolution, where human-controlled artificial selection replaces natural selection [65]. Domesticates, therefore, provide good opportunities to test the possibility that environmentally induced epimutations may end up as genetic variants. Support towards this possibility is available for domestic mammals [66], birds [67] and fishes [63]. Thus, an increasing number of observations suggest that environmentally induced epimutations may end up fixed as genetic modifications, provided the environmental change persists across a sufficient number of generations (figure 2*b*). Importantly, in this scenario, a given environmental cue can alter the DNA methylation of a given loci de novo in each generation in what is termed ‘epigenetic wash-in’ [68]. If these epimutations are persistent enough, and thanks to their ability to induce mutations, these mutations could eventually increase their frequency in the population and constitute the genetic basis for a new phenotype [64].

## 4. A proposal: a role for epigenetics in regulating sex fate and plasticity

**Figure 3.**
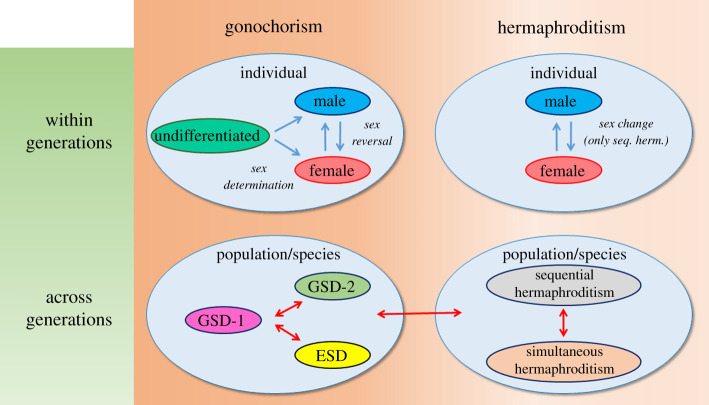
Summary of the proposal made in this review: epigenetic mechanisms have two main roles in the regulation of vertebrate sex. First, within one generation and at the individual level, by contributing to the acquisition of the male *or* female phenotype during sex determination/differentiation in gonochoristic species, or the acquisition of the male *and* female phenotypes at the same time in simultaneous hermaphrodites, or first as one sex and then as the other in sequential hermaphrodites (seq. herm.). In the latter, they are involved in the process of sex change and, in gonochoristic species, in sex reversal, if that happens to occur (e.g. owing to environmental perturbations during early sensitive periods). Second, across generations, by promoting the evolutionary transitions between one GSD system to another (GSD-1 to GSD-2) or to ESD (GSD-1 to ESD), and between any of these sex determining systems to the different forms of hermaphroditism. As shown in this review, there is evidence supporting the implication of epigenetics in developmental processes (blue arrows) but further evidence is needed to clearly support evolutionary transitions (red arrows).

Based on accumulating evidence and on what has been discussed in the previous sections, epigenetic mechanisms may have two main roles, at least in vertebrate sex. First, and within a given generation, by contributing to the acquisition and maintenance of (i) the male *or* female function once during the lifetime in individuals of gonochoristic species, and (ii) the male *and* female function in the same individual, either at the same time in simultaneous hermaphrodites, or first as one sex and then as the other sex in sequential hermaphrodites. Second, if environmental conditions change, epigenetic mechanisms may also have a role by providing the necessary phenotypic plasticity to facilitate the transition (i) from one sexual system to another, or (ii) from one sex-determining mechanism to another. Furthermore, if the environmental change lasts enough time and there is transgenerational inheritance, epimutations could facilitate assimilation into genetic changes that stabilize the new sexual system or sex-determining mechanism. There would be, therefore, two temporal axes for the action of epigenetic mechanisms regulating sex: one within the same generation, contributing to the acquisition and maintenance of sex; and another across generations (figure 3).

Regarding the main first role, i.e. sex determination and maintenance, epigenetic mechanisms would then contribute, in a given individual and regardless of whether the species to which that individual belongs is gonochoristic or hermaphroditic, to the regulation of the gene expression program necessary for sexual fate commitment. Accumulating evidence supports the involvement in this first role, as outlined above, and to gather additional evidence research in more species should be carried out. Thus, to fulfill key knowledge gaps, we have that in species with ESD, there is a testable hypothesis on how the environmental cue can be captured and the signal transduced by cellular signalling pathways and epigenetic processes, the CaRe hypothesis [57]. Further, in both GSD and ESD gonochoristic species, as well as in hermaphroditic species, there is the testable CERS model on the sex-specific relationship between epigenetic activation or repression and gene expression concerning both pro-male and pro-female genes of the network [52]. Both models provide a framework to advance our further understanding. Studies aimed at confirming the role of epigenetics in sex acquisition and maintenance should attempt to find: (i) mechanisms linking environmental perturbations to epigenetic changes by the analysis of molecules and signalling pathways; (ii) finding functional consequences of epigenetic modifications, i.e. a sex being associated with a given set of epigenetic marks, as has it already been demonstrated in the European sea bass [35]; and (iii) determine whether epigenetic changes are cause or consequence of a given gene expression programme. To this end, help can come from manipulations of the epigenome by the use of DNA methyltransferase inhibitors such as 5-aza-2′-deoxycytidine (5-aza-dC), shown to be able to alter sex ratios in zebrafish, *Danio rerio* [69], or the more recently developed technique to edit the methylome in the mammalian genome [70]; and (iv) dealing with the fact that epigenetic marks mostly are cell specific. Thus, cell-specific analysis approaches, currently becoming more used, are needed. Of note, besides the importance that one may give to the epigenetic regulation of sex, it should not be forgotten the crucial role of transcription factors such as Sox9, Foxl2 and Dmrt1 in sex determination and maintenance [50]. Thus, epigenetic mechanisms contribute and are necessary but not sufficient for the establishment of sex.

Epigenetic differences between sex chromosomes are being found as the number of species being examined increases, as shown above. More research in the role of epigenetics in sex chromosome evolution is needed, and the contribution of DNA methylation in recombination suppression is compatible with the recent view that sex chromosome evolution is not necessarily a simple progression of accumulating divergence [33].

Testing the second main role, i.e. the involvement of epigenetics in evolutionary transitions is more challenging and here there is an important knowledge gap. Deciphering the contribution of epigenetics in evolutionary transitions can benefit from ongoing efforts aiming at elucidating whether epigenetic modifications can facilitate the inheritance of novel phenotypic variants that are generated by environmental change, a strategy called ‘heritable bet hedging’ [60]. Of note, environmentally induced epigenetic changes can also produce heritable maladaptive phenotypes, a phenomenon termed ‘epigenetic traps’ [60]. In this regard, it has been argued that epigenetically mediated alterations in sex ratios could become an epigenetic trap in ESD species facing rapid climate change by consistently producing heavily skewed sex ratios ([71]; but see also [72]).

To test the role of epigenetics in evolutionary transitions between sexual systems, taxa where the sexual system varies by order, family, genus or even species, such as Cnidaria, polychaetes and teleost fishes [2] would be most appropriate. The use of techniques such as WGBS could allow the identification of differentially methylated cytosines or differentially methylated regions in the gonads (provided they are examined at exactly the same developmental time and stage of the reproductive cycle) in key genes involved in sex determination. Closely related and sympatric species with contrasting sexual systems such as, for example, *Diplodus puntazzo* (gonochorist) versus *Diplodus annularis* (protandrous) [73], would be appropriate.

Recently, the assessment of patterns in the evolution of sex-determination systems in the diverse vertebrate clades of teleost fishes, squamate reptiles and amphibians evidenced not only similar transition rates between homomorphic and heteromorphic sex chromosomes in both fishes and amphibians but also to ESD from heteromorphic versus homomorphic sex chromosome systems in fishes [5]. These observations would then not support the view that heteromorphic sex chromosomes can be a sort of ‘evolutionary trap’ [74]. Thus, to test the role of epigenetics in evolutionary transitions between sex-determining systems, taxa where frequent transitions between sex-determining systems occur can be useful.

In this regard, in amphibians, the ancestral system is thought to be ZW/ZZ with multiple transitions to XX/XY. Transitions are frequent and can be seen in ‘real time’, as some populations of *Glandirana* (formerly *Rana*) *rugosa* from Japan have a male heterogamety (XX/XY) while others have female heterogamety (ZW/ZZ), with different degrees of transition. It is believed that the transition from the ZW/ZZ system to a XX/XY has occurred at least twice independently [75,76]. Theoretical models predict homology between the W and X chromosomes and the Y and Z chromosomes, and it has been suggested that the dominant master sex-determining gene of one heterogametic system could be the dosage-dependent master gene in the other [77]. Analysing DNA methylation in the sex chromosomes and key genes of the sex-determination cascade would provide insights on the role of epigenetics in the regulation of gene expression in these contrasting sex-determining systems. Not only species with chromosomal sex determination (CSD) would be appropriate. Species with PSD in a group of species with CSD could also be a good model to test evolutionary transitions from one genetic sex-determination system to another, where the coexistence of newly emerged and ancestral sex-determining genes would be expected. For example, it would be worth exploring whether observed differences in the evolutionary trajectories and expression levels of aromatase genes in African cichlids, [78] are related to changes in epigenetic transcriptional regulation.

Finally, the detection of epigenetically facilitated mutations is challenging and will need specific experiments [64]. Epigenetic changes must occur in the germline for any evolutionary significance. Among vertebrates, fishes may have an advantage as they seem to show less reprogramming of epigenetic marks during gametogenesis when compared to mammals [35,79–81], facilitating the transmission of epigenetic marks from parents to offspring. Inheritance of epigenetic marks across one or more generations in association with the influence of environmental factors such as hormones [82], hypoxia [83] and temperature [84] has been reported. In this regard, it is worth noting that exposure of zebrafish to elevated temperature is able to induce sex ratio shifts and alterations in the testicular epigenome of the unexposed offspring [85].

The eventual identification of differences in the role of epigenetics in sexual fate, plasticity and evolution across large taxa perhaps could contribute to shed light on why certain phyla and classes are quite labile and the sexual system varies even within a genus, in some cases, whereas in others are very rigid with little or no diversity. Thus, the large-scale distribution of sexual systems is best explained by phylogeny rather than by sex allocation theory, a situation known as Williams' paradox [2,4]. Although phylogenetic differences in epigenetic regulation have been reported [86], whether this affects sex determination and sexual systems is at present unknown.

## Data accessibility

This article does not contain any additional data.
